# Enhancing COVID-19 Vaccine Uptake among Tribal Communities: A Case Study on Program Implementation Experiences from Jharkhand and Chhattisgarh States, India

**DOI:** 10.3390/vaccines12050463

**Published:** 2024-04-26

**Authors:** Ankita Meghani, Manjula Sharma, Tanya Singh, Sourav Ghosh Dastidar, Veena Dhawan, Natasha Kanagat, Anil Gupta, Anumegha Bhatnagar, Kapil Singh, Jessica C. Shearer, Gopal Krishna Soni

**Affiliations:** 1 PATH, Seattle, WA 98102, USA; jshearer@path.org; 2 John Snow India Pvt. Ltd., Delhi 110070, India; manjula.sharma1972@gmail.com (M.S.); tanya.25@gmail.com (T.S.); gdsourav@gmail.com (S.G.D.); gupta.dranil@gmail.com (A.G.); anumegha_bhatnagar@in.jsi.com (A.B.); sonigk70@gmail.com (G.K.S.); 3 Ministry of Health & Family Welfare, Government of India, New Delhi 110011, India; veena.dhawan@gov.in; 4 John Snow Inc., Arlington, VA 22202, USA; natasha_kanagat@jsi.com; 5 World Health Organization, New Delhi 110011, India; singhkap@who.int

**Keywords:** tribal populations, COVID-19 vaccination, India, implementation science, case study, community-based strategies

## Abstract

Tribal populations in India have health care challenges marked by limited access due to geographical distance, historical isolation, cultural differences, and low social stratification, and that result in weaker health indicators compared to the general population. During the pandemic, Tribal districts consistently reported lower COVID-19 vaccination coverage than non-Tribal districts. We assessed the MOMENTUM Routine Immunization Transformation and Equity (the project) strategy, which aimed to increase access to and uptake of COVID-19 vaccines among Tribal populations in Chhattisgarh and Jharkhand using the reach, effectiveness, adoption, implementation, and maintenance framework. We designed a qualitative explanatory case study and conducted 90 focus group discussions and in-depth interviews with Tribal populations, community-based nongovernmental organizations that worked with district health authorities to implement the interventions, and other stakeholders such as government and community groups. The active involvement of community leaders, targeted counseling, community gatherings, and door-to-door visits appeared to increase vaccine awareness and assuage concerns about its safety and efficacy. Key adaptations such as conducting evening vaccine awareness activities, holding vaccine sessions at flexible times and sites, and modifying messaging for booster doses appeared to encourage vaccine uptake among Tribal populations. While we used project resources to mitigate financial and supply constraints where they arose, sustaining long-term uptake of project interventions appears dependent on continued funding and ongoing political support.

## 1. Introduction

Tribal populations make up roughly 8% (104 million people) of the Indian population and comprise 700 scheduled tribes [1,2]. Tribal populations in India are heterogeneous, distinct in language, culture, and belief systems. Nearly 90% of all Tribal populations live in rural areas, often in dense forests which make it hard to access and deliver health services [1]. About 45% of all scheduled tribes, which have been acknowledged as among the most disadvantaged socio-economic groups in India, fall below the poverty line and have literacy rates that are persistently lower than the national average [1]. Furthermore, several studies on Tribal populations’ health care preferences have highlighted their lack of trust in medical treatments or prevention measures and their preference for traditional medicines, practices, [3] and healers [4,5]. Socioeconomic differences, inadequate access to health care, geographic remoteness, cultural practices, and unique perspectives on health contribute to disparities in health outcomes among Tribal populations, compared to the general population [3].

Furthermore, connection with one’s community appears to be central to Tribal populations. This connection fosters the strong sense of belonging that has been observed among tribal and indigenous populations living in India [6], Australia [7], and the United States [8]. Consequently, tribal populations often prefer to seek care from traditional providers within their community, as noted also in Bangladesh [9]. Strategies that employ individuals within Tribal communities to deliver health services, and leverage the social capital of key opinion leaders, such as village leaders and community health workers from those communities, have been shown to bridge gaps with the local health system in India [10]. Broader community participation has also been considered to be critical for expanding access to services [6]. Despite these distinct characteristics and preferences, India’s health system has taken a uniform approach to Tribal and non-Tribal population health, with inconsistent attention paid to the differences in economic and educational attainment, language, geographic access to health professionals, and cultural beliefs that shape care-seeking behaviors [6]. A 2021 UNICEF study from India citing 2015–2016 NFHS-4 data noted that only 56% of Tribal children were fully vaccinated (compared to 62% nationally) [11]. Tribal populations also have disproportionately higher rates of diseases such as malaria, leprosy, and tuberculosis, and higher rates of malnutrition than the non-Tribal population [3]. The under-five mortality rate among scheduled tribes is 50 per 1000 live births; the national average is 42 per 1000 live births [12].

Tribal populations’ health challenges were exacerbated during the pandemic. India introduced COVID-19 vaccines in January 2021 [13], and in the early stages of the vaccination campaigns that year, the national government initially reported that 72% of Tribal districts were surpassing the national average in terms of vaccination coverage [14]. However, coverage disparities quickly emerged between Tribal- and non-Tribal-majority districts. The lowest coverage was observed in Tribal districts in the Northeast, followed by those in Jharkhand and Chhattisgarh [15]. In October 2021, 63% of districts with Scheduled Tribe populations exceeding 20% were falling behind the national average of 53% of people having received at least the first dose of the vaccine [14,16]. Lower vaccination coverage among Tribal populations was attributed to lack of awareness [15] and misinformation about COVID-19 [17]; overburdened community health workers having to travel longer distances to reach Tribal populations [18]; and fluctuations in vaccine supply [15].

MOMENTUM Routine Immunization Transformation and Equity (the project) began working across 18 states in India to catalyze the government’s efforts to increase COVID-19 vaccine coverage among priority populations. Increasing vaccine uptake among Tribal populations was a key focus for the project in Chhattisgarh and Jharkhand, where roughly one-third and one-quarter of the population, respectively, identify as scheduled tribes [19]. In collaboration with the state government, the project partnered with two community-based nongovernmental organizations (CBOs)—Samarthan in Chhattisgarh and the Indian Society of Agri-Business Professionals in Jharkhand—to increase awareness and vaccination uptake among Tribal populations, beginning in January 2022.

The project entered the two states after the initial COVID-19 vaccination rollout by the government, and at a time when resources from the COVID-19 program were diminishing and significant human resource capacity gaps were emerging. The project was positioned to address these gaps by creating awareness and demand among Tribal populations who remained unvaccinated, in collaboration with the CBOs and the government.

Based on a series of formative discussions with Tribal community members, community health workers, and government staff, the project began uncovering the multiple reasons why some Tribal populations remained unvaccinated. Some strongly believed that COVID-19 was not a major concern, as they had not witnessed significant illness in their communities. This led to a strong sense of complacency in Tribal communities, particularly among elderly members, who felt that death was imminent and therefore did not seek vaccination. Younger Tribal members were concerned about potential side effects from vaccination, particularly infertility. Even among those who were vaccinated, there seemed to lack a clear understanding of the benefits of vaccination. However, social factors, such as the influence of family members, did motivate some individuals to receive the vaccine. There were instances where unvaccinated elders, despite their own complacency, encouraged their family members to get vaccinated to prevent illness. Tribal populations also reported trusting those within their community, such as their community health workers and village leaders, for advice on health issues such as vaccination. Receiving the COVID-19 vaccination certificate, which at the time was a requirement for travel and work, was also seen as an important motivator.

Taking account of these factors, the project implemented four key interventions to increase vaccine uptake among Tribal populations across both states (Table 1). These interventions focused on: (i) developing strong partnerships with government and frontline community health workers to identify the unvaccinated and arrange vaccination sessions; (ii) collaborating with trusted community members from the Tribal community to expand outreach within the Tribal community; (iii) conducting interpersonal outreach and awareness campaigns by hiring Tribal community members who speak the local language and can build trust with unvaccinated Tribal members; and, more broadly, (iv) leveraging community events to raise awareness and encourage vaccine uptake using existing forums.

In this paper, we applied the reach, effectiveness, adoption, implementation, and maintenance (RE-AIM) framework to assess the success of the project’s interventions for increasing vaccine uptake among Tribal populations [20]. We conclude by reflecting on how these insights might inform the design and implementation of interventions for other health programs for Tribal populations.

### Overview of Conceptual Framework

Applying the RE-AIM framework was relevant in our case study, where interventions are complex and may enable a deeper understanding of various outcomes, including unintended benefits and consequences. This approach not only enhanced our understanding of the project interventions’ effectiveness, but also of the mechanisms behind the outcomes, accounting for external factors influencing implementation, adoption, long-term sustainability, and potential scale [21].

Table 2 provides the definitions of the RE-AIM framework dimensions examined in our study. We adapted these definitions to align with our study context. For the implementation dimension, instead of primarily assessing fidelity to a rigid intervention design, we modified the definition to recognize and capture adaptations made to the interventions for the unique Tribal sub-contexts in which the project was implemented.

## 2. Materials and Methods

A. Study Design

We designed a qualitative, retrospective, and explanatory case study guided by the RE-AIM framework to assess the success of the project strategies. Because the project strategies collectively aimed to increase COVID-19 vaccination uptake among Tribal populations in both states, our evaluation centers on the project strategies as a unified case, rather than separately assessing each intervention. This approach also acknowledges the interdependence of these project strategies. Therefore, the case is the implementation of project strategies, and the unit of analysis is the community covered by the project. A case study approach was considered appropriate because it can help to answer not just what happened, but why and how it happened within the complex and evolving contexts where the case was unfolding [22].

This case study conducted qualitative in-depth interviews (IDIs) or focus group discussions (FGDs) with three key respondent groups: (1) Tribal populations that interacted with the project’s activities; (2) CBOs that designed and implemented project interventions; and (3) key community-, district-, and state-level stakeholders who supported the implementation of project interventions. The interview and discussion guides were informed by the RE-AIM framework.

B. Study Context

Data collection took place in two districts each in Jharkhand and Chhattisgarh. District selection was determined based on two criteria: (1) whether the district was an M-RITE project district; and (2) whether it had a significant Tribal population, so that we could glean insights into how the project strategies were working. According to these criteria, data collection was conducted in Giridih and West Singhbhum in Jharkhand, and Gariabandh and Kanker districts in Chhattisgarh.

In Chhattisgarh, the project implementation in both districts began in October 2021, and implementation concluded in January 2023 in Kanker, and June 2023 in Gariabandh. Project implementation in both districts of Jharkhand started in March 2022 and ran until January 2023 in Giridih, and June 2023 in West Singhbhum.

Based on the most recent district-level census data available on Tribal populations [2], West Singhbhum and Giridih in Jharkhand, respectively, have Tribal populations of 1,011,296 and 238,188. Kanker and Gariabandh districts have populations of 414,770 and 173,977, respectively. The Tribal communities in Jharkhand and Chhattisgarh are linguistically diverse and live in densely forested areas. Some rely on foraging in the forests for sustenance, while others engage in various occupations such as agricultural labor, iron smelting, rope making, and household industries [23]. Each Tribal community is tightly knit, and their village and community leaders can have a significant influence on their decision-making. In this context, CBOs became instrumental project partners; they came with a deep understanding of the local context and used their existing infrastructure and community networks to develop and implement the project strategies outlined in Table 1. Their operational structure in the project involved a decentralized network of district coordinators, block coordinators, community supervisors, and vaccine ambassadors, or dedicated village volunteers. They also collaborated with grassroots community groups to further enhance their reach in Tribal communities.

C. Data collection

### 2.1. Tribal Populations

The IDIs and FGDs with Tribal populations aimed to identify factors influencing COVID-19 vaccine uptake for these populations and if and how the project’s strategies contributed to it. We conducted separate FGDs with men and women, and IDIs with Tribal population members when one-on-one translators were needed. We attempted to conduct interviews with both vaccinated and unvaccinated individuals but given the advanced stage of the COVID-19 vaccination program in the states, most of our respondents were at least partially vaccinated. Across both states, 17 focus group discussions (FGDs) and 21 IDIs were conducted with Tribal community members.

### 2.2. Community Based Organizations

We conducted 5 FGDs with CBO staff at the district, block, and community levels in both states to understand the designs of project interventions, reach, effectiveness, and implementation experiences, including the barriers to and facilitators of their implementation, and adaptations made along the way to increase COVID-19 vaccine uptake in Tribal populations. We conducted 6 IDIs with additional community-based CBO staff to gain deeper insights into project implementation experiences.

### 2.3. Key Stakeholders

Across the two states, we interviewed 12 community-level stakeholders, such as village heads, vaccine ambassadors, and community health workers, at the block and district levels to better understand their roles and responsibilities in implementing the project strategies. We also sought their perspectives on the reach and effectiveness of the strategies and their experiences interacting with the project. Additionally, we interviewed a total of 29 district- and state-level stakeholders who were asked to reflect on the same issues and were further questioned about potential mechanisms for sustaining the project’s strategies. District-level government stakeholders included members of the district-level COVID-19 task force, and district health officials. State-level respondents included representatives from United Nations organizations, government officials, and National Health Mission staff.

In total, we conducted 68 IDIs and 22 FGDs across the 2 states (Table 3).

All of the qualitative data were collected in person from December 2022 to April 2023 in Jharkhand and Chhattisgarh. IDIs usually lasted 30–45 min, and FGDs 45–105 min. All participants were at least 18 years old. Oral informed consent was obtained from all the participants and conversations were audio recorded with permission. An agency transcribed all the conversations verbatim, and the research team reviewed the transcriptions for quality.

D. Data analysis and validation

We conducted a thematic analysis using the five dimensions of the RE-AIM framework [19]. We extracted textual data from the IDIs and FGDs into a spreadsheet with the categories from the analytical framework. Following this, we developed memos that summarized the information by category and compared and contrasted findings by district and state. While writing the memos, we triangulated data between FGDs and IDIs according to the three respondent categories highlighted in Table 3. We debriefed following each step to deepen our understanding of the data and conducted a meeting with a few respondents to validate study findings. We also conducted a learning workshop in October 2023, through which nearly 70 project participants were brought together, including CBOs, government partners, and state-level project staff, to review and validate the findings and conclusions. In the workshop, we also discussed how the learnings might be applied to other health programs. We also triangulate the qualitative findings of this study, where possible, with quantitative project data, which is routinely collected by the CBOs working at the district level in both states.

## 3. Results

Since there were no major differences in project strategies between the two states or by district, our findings are presented collectively across the RE-AIM domains of reach, effectiveness, adoption, implementation, and maintenance.

### 3.1. Reach

Most of the qualitative data suggest that the project’s strategy to collaborate with trusted community stakeholders such as village heads, the project’s village ambassadors, and community health workers succeeded in reaching Tribal populations in the project intervention districts in Chhattisgarh and Jharkhand and creating general awareness about vaccination sessions. Specifically, CBO project implementers said that the project strategy helped them gain trust and build rapport with Tribal populations and increased community-member participation in activities.

Tribal members whom we interviewed consistently said they viewed village heads, who make announcements in the evenings when everyone has returned from work, as the primary source of information about awareness activities and vaccination sessions. One district-level government staff worker agreed that the nominated village heads had a strong role in creating awareness of the COVID-19 vaccines, stating that “most effective were the words of *munda* [village head] only” [IDI-07, district-level stakeholder, Jharkhand]. In Chhattisgarh, some Tribal population members described learning about vaccination sessions through WhatsApp group messages sent by the members of the *panchayati raj*, an assembly of the village government. According to CBO staff, involving trusted community stakeholders who speak the local language helped them convey the benefits of vaccination more clearly.

Community health workers, particularly those in Jharkhand, were also considered critical information sources by the Tribal community. A community health worker described how she helped CBOs connect with Tribal populations, who generally tend to be wary of outsiders:

“When someone from the village stays in the meeting, then the villagers feel comfortable, otherwise the outsiders have to face some trouble. So, if we are present in any meeting either I [auxiliary nurse midwife] or a *sahiya* [accredited social health activist, a community health worker]… the meeting goes smoothly”.

[IDI-02, community health worker, Jharkhand].

CBO staff similarly found that collaborating with community health workers helped them reach the Tribal community.

In addition, CBO staff perceived their efforts to partner with local organizations, including SHGs and *yuva mitan* (youth groups) clubs, to be important for increasing the Tribal community’s awareness about vaccination initiatives. These groups encouraged participation in *ratri chaupals* (evening community gatherings), and appeared to increase community awareness of vaccination sessions through word-of-mouth.

For example, in Chhattisgarh, 24 SHG members motivated people within their Tribal community to get vaccinated. They formed groups of 3–4 and visited people in their fields and farms during work hours to increase awareness about the COVID-19 vaccination session. During these visits, SHG members described their personal experiences of receiving the vaccine and highlighted remaining in good health after vaccination to reassure others about the vaccine’s safety. One SHG member said she tried to lead by example when encouraging others to receive the vaccine: “I had to first get vaccinated before I tell others to go for vaccination.” [FGD-03, key stakeholder, Male Tribal population member, Chhattisgarh].

Youth groups helped organize *ratri chaupals*, and became regular facilitators at these events in Chhattisgarh. A total of 70 *ratri chaupals* were implemented in the two districts of Chhattisgarh. CBO staff said that involving youth and children in *nukkad natak* (street plays) during these gatherings injected new energy into the vaccination messages. More broadly, Tribal and CBO respondents said that the timing and structure of *ratri chaupals* increased the project’s reach and that these gatherings were well attended.

Based on program data, overall, the project interventions reached 212,679 individuals in Giridih, Jharkhand, and 80,478 individuals in West Singhbhum, Jharkhand, during the project period. Similarly, the project reached 153,498 individuals in Gariabandh, Chhattisgarh, and 210,696 individuals in Kanker, Chhattisgarh.

### 3.2. Effectiveness

Considering the project’s strong collaborations with the government, local organizations, and community health workers, and the implementation of a “whole-of-government strategy” during the COVID-19 vaccination rollout [20], assessing the effectiveness of the project’s strategies alone was challenging. In this section, we evaluate the effectiveness of the project’s strategies in increasing demand for, access to, and uptake of the COVID-19 vaccines among Tribal populations, but acknowledge that it was influenced by broader government policies and practices across the two states.

Overall, our interviews indicate that the project’s four strategies appeared to enhance Tribal populations’ access to, demand for, and uptake of COVID-19 vaccines. We found that the project’s strategy of collaborating with trusted community stakeholders not only expanded its reach among Tribal populations, as described in the section above, but also generated demand and uptake among them. One CBO respondent mentioned that announcements from village leaders and their messengers (a project intervention) established the trust necessary to enhance vaccine demand and uptake:

“We have appointed a community supervisor which is a munda [an unelected community/village head]… and belongs to the same Tribal community, same society so whenever he communicates then the people do agree and get vaccinated”.

[IDI-10, district coordinator, CBO, Jharkhand].

Tribal community members also acknowledged that the village head’s recommendation to get vaccinated was perceived as a directive, which increased their intention to get vaccinated, with one individual saying, “The *sarpanch* [elected village head] said that my name has been identified for vaccination… It’s a government order.” [FGD-01, key stakeholder, Tribal population member, Chhattisgarh].

Another Tribal population member described how village heads were informed by community health workers about individuals who were or were not vaccinated, creating a sense of urgency:

“Sahiya [community health worker] tells *dakua* [a village leader] if someone goes to *Panchayat Bhavan* [village center] and is not taking the injection. Then, dakua in morning time does the announcement [about the next vaccination session]”.

[FGD-01, key stakeholder, Female Tribal population member, Jharkhand].

Similarly, the CBOs and community members said that hiring vaccine ambassadors or volunteers from within the community (a project intervention) facilitated Tribal populations’ access to vaccination. The project trained and engaged a total of 655 volunteers in the two districts of Jharkhand and 1017 in the two districts of Chhattisgarh. Tribal community members highlighted how project volunteers and government community health workers accompanied them to the vaccination sites using a project vehicle. One Tribal community member commented on the effectiveness of this intervention, stating:

“The project made it easy to access the vaccine. However, there were long vaccine queues, but everyone was there so we lined up, too”.

[FGD-07, key stakeholder, Male Tribal population member, Jharkhand].

Engaging organizations rooted in the Tribal community was critical to mobilization. The community trusted them, especially when they assured people as to the absence of potential adverse effects resulting from the vaccine:

“So, we had to take the help from the head of the *gram panchayats* [village council] and lead members of the SHGs to convince people to take the COVID vaccine. We organized camps in villages to roll out the COVID vaccines among people. I convinced people to take the COVID vaccine by giving them my example and telling them that nothing would happen to them if they took the vaccine. So, I was able to convince 10 people to change their mind and take the COVID vaccine. I made a list containing the details of those 10 people and gave them to one of our nurses”.

[IDI-05, local CBO, Chhattisgarh].

The use of interpersonal communication activities like door-to-door visits by the project’s community volunteers/vaccine ambassadors and community health workers (project intervention) appeared to raise awareness about the importance of vaccination and motivate vaccination, especially among Tribal community members who were very hesitant. One couple described fear of vaccination and said that a neighbor’s visit and door-to-door visits by the project staff and a government community health worker were motivating:

“So, after listening to them [community health worker and project staff] only they agreed. Like it happens that they will listen to only their caste people, they will do if they say or else they will not… In terms of this vaccine, it was the same way. They did listen to what the *sevika and sahiya* [community health workers] said”.

[IDI-01, key stakeholder, PVTG Tribal population member, Jharkhand].

Another Tribal community respondent, initially hesitant about vaccination due to safety concerns, also said that the door-to-door visit motivated vaccination: “People from an organization came to tell us that taking the corona vaccine is necessary, and it doesn’t cause fever, so we got vaccinated.” [FGD-05, key stakeholder, Female Tribal population member, Jharkhand]. This respondent also acknowledged that hearing about vaccination from different sources, including community health workers, village leaders, government officials, and the project, helped them trust the vaccine and be more comfortable with vaccination. In some cases, the project’s interpersonal communication activities seemed to contribute to vaccine uptake, although data from the interviews indicating whether uptake was a direct result of the project strategy or other reasons are limited.

While one-on-one interactions were effective in some settings, other respondents described receiving the vaccination without much explanation. One Tribal respondent indicated that the project staff met with her and other women and a community health worker, but “we did not get an explanation of why we need to get it. We got it because the *sahiya* [community health worker] was upset.” [FGD-01, key stakeholder, Female Tribal population member, Jharkhand]. This suggests that despite the project’s strategy to actively listen and respond to Tribal population member concerns, social pressure may have influenced vaccination, too.

The project’s use of government health and non-health sector platforms to enhance the awareness of COVID-19 vaccines seemed effective. As one Tribal respondent stated:

“Everyone around was telling us to get vaccinated. The sarpanch, the *kotwar* [village announcer], everyone was recommending vaccination. The government was also ordering everyone to get the vaccine. If they said to do it, you have to do it”.

(05, Tribal population community member, Chhattisgarh).

CBO respondents also felt that this strategy may have contributed to increased vaccine uptake among Tribal members who attended those events, but there is a limited amount of data as to whether or how this strategy directly influenced uptake.

### 3.3. Adoption

Our interviews with district-level CBOs and government officials suggested that there were no major barriers to adopting the project strategy, given that it was developed at that level, and collaboratively. However, interviews with government health workers and CBO staff at the block and community levels indicated delayed adoption and implementation of certain interventions, particularly door-to-door visits and vaccination sessions, due to community health workers’ initial reluctance to assist.

At first, many community health workers who were expected to support CBOs in the implementation refused to participate because they did not receive the government-promised financial incentives for conducting COVID-19 vaccine-related activities during the initial rollout. Over time, most community health workers supported and developed trusted partnerships with the CBOs because they saw value in them, particularly as CBO staff provided transportation to conduct vaccination activities and supported them with routine tasks such as data entry and microplanning. One CBO staff member acknowledged this support, saying, “Community health workers have not been paid anything for 2–3 years, but they are still working with us.” [FGD-02, CBOs, Jharkhand]

More broadly, some project stakeholders acknowledged that district-level CBO project staff appeared to motivate and energize the government community health workforce.

### 3.4. Implementation

Though the overarching project strategies were implemented in both states, flexibility was essential to allow adaptations to overcome Tribal populations’ most prominent barriers to vaccine demand, access, and uptake at any given time.

That said, internal project factors such as staff attrition and external contextual factors, including health system strength, affected the project’s ability to implement the strategies as intended. This section describes the facilitators of and barriers to implementation, and the adaptations to improve effectiveness. Table 4 provides a summary.

#### 3.4.1. Internal Factors

As to the barriers to implementation, staff attrition hindered the implementation of the project’s strategy. Staff turnover, which was primarily due to poor performance, resulted in frequent recruitment of new block-level coordinators. Attrition was also noted to be higher in areas that were considered sensitive due to the presence of certain militant groups commonly referred to as Naxalites. To address this gap, quick hiring processes were implemented, but recruitment times were often lengthy. One CBO also implemented an “all hands-on deck approach” during which the entire district-wide project workforce conducted vaccine mobilization activities in every village of the district (described further in Section 3.4.3, Adaptations to Project Interventions). In addition, some CBO staff noted varying levels of experience among their coworkers, ranging from former university professors to those learning on the job. These varying backgrounds at times facilitated knowledge sharing and learning. Despite these barriers, teams across both states acknowledged that working within supportive teams at the block and district levels facilitated quick implementation.

##### Facilitators

Two internal factors facilitated project implementation. First, strong support from the project’s state leadership, which conducted regular meetings with CBO staff and teams that had trouble increasing vaccine uptake, helped troubleshoot problems quickly and effectively. One district-level CBO staff member described this approach, saying, “The state coordinator holds daily meetings in areas where teams were facing difficulties and creates microplans.” (FGD-02, CBOs, Chhattisgarh)

CBO staff said that this learning-by-doing approach guided program implementation and problem-solving, and improved their understanding of what did and did not work. Beyond these meetings, the state and district coordinators visited local teams to overcome challenges and co-develop solutions. Together they conducted FGDs with community members, held conversations with panchayati raj and village leaders, and sought feedback from district government leaders, which helped them understand how their strategies were working.

The second facilitator was hiring local individuals with networks and experience, which CBO staff stated accelerated implementation and activities, particularly at the block and community levels. They were viewed as trusted insiders who knew how to navigate cultural sensitivities and adapt their communication to be culturally responsive.

#### 3.4.2. External Factors

##### Barriers

A few external barriers affected the quality of project strategy implementation in the two states. First, the government’s vaccine wastage policy significantly influenced the timing and locations in which vaccines were offered and administered to Tribal populations. Because districts enforced strong policies to minimize wastage, CBO and government community health workers explained that gathering enough vaccine recipients from Tribal communities was a logistical problem. Though vaccination sessions were organized for 10 people, if fewer arrived, health workers refrained from opening the vaccine vial until the tenth person arrived. As one Tribal community member said, “they [health worker] don’t open it unless 10 candidates are present. Not even if 9 are present, they would wait for one more person to come.” [FGD-03, Tribal population, Chhattisgarh].

CBOs staff explained that this policy led to situations in which some Tribal population members, despite attending a session, were turned away without getting vaccinated. In some cases, there was immediate coordination with another district or block to identify more eligible people for vaccination. However, this meant that people already at the vaccination session had to wait until more people arrived before vaccination could begin, which lowered the overall quality of service experience.

Second, CBO staff and health workers described broader vaccine shortages in the state, which were seen as a major barrier to vaccine mobilization and uptake. As one staff member said:

“We are facing an issue with the vaccine availability. This impacts our mobilizing activity because people would say that a team came telling us about the vaccine but when we reached, there was no vaccine available. We are feeling bad to mobilize now because even if we mobilize, they won’t get vaccinated at the end. People are missing out doses during vaccination camps because of unavailability of vaccine taken at first dose”.

[FGD-02, CBO, Jharkhand].

Some CBO respondents indicated that they helped transfer excess supply of vaccines to blocks or districts with low or no inventory:

“There was an instance of a vaccine camp where… there was no medicine and in the other village no one came to administer the vaccine. We immediately arranged for vehicles to transport the vaccines from one location to the other”.

[IDI-04, CBO, Chhattisgarh].

Third, unpaid government data entry operators at the block and district levels resulted in a backlog of individuals awaiting data entry into Co-WIN, the national COVID-19 vaccine registration and reporting platform, in both states. This backlog hindered the generation of vaccination certificates (a key motivator of vaccination); the timing of the second vaccine dose for many individuals; and the preparation of lists of individuals who were due for vaccination. The CBOs and government community health workers used these data to determine the locations of their door-to-door visits, *ratri chaupal*, and, subsequently, the vaccination sessions. To alleviate the problem, CBOs supported data entry and hired temporary data entry staff at the block and district levels.

Fourth, resource constraints hindered intervention implementation. Transportation funds provided by the government to community health workers to conduct awareness and mobilization activities were quickly exhausted during the initial COVID-19 vaccine rollout. The absence of these funds made it difficult for health workers to accompany CBO staff on door-to-door activities, especially in distant and politically sensitive blocks, which generally have a higher density of Tribal population. The project stepped in to provide transportation to frontline community health workers, which was a key motivator for collaborating with CBO staff.

##### Facilitators

The “whole of government” approach to COVID-19 vaccination helped the project to benefit from high levels of political will within the health department and increased access to the various departments that were all working to increase vaccine uptake.

Specifically, strong political will and the demand to increase vaccination coverage increased the intensity of project implementation activities in the districts. The district magistrates held meetings to review and monitor vaccine data closely, creating a high level of accountability that was critical to achieving vaccination targets; as one CBO staff member stated, ‘’If the district magistrate is strict, then the district’ coverage is higher.” [IDI-16, district-level stakeholder, Chhattisgarh].

The heightened accountability facilitated coordination and collaboration across various departments, and allowed the project team to access names and contact information of Tribal members eligible for the government’s supportive services. CBOs cross-referenced the names of members listed in other departmental programs to develop a more comprehensive list of people due for vaccination and prioritize Tribal villages with low vaccination coverage for community awareness activities.

Second, as stated previously, partnerships with the government, particularly community- and block-level health workers, was critical for project implementation because of their relationships with Tribal populations and key leaders and their connection to the health system:

“We couldn’t have visited these PVTG [Tribal] villages without the *mitanins* [community health workers]… who proved to be quite helpful for us because they are local people who know what goes on in their village. They provided us with staff and adequate vaccines to give to people. They informed the RHO and CHO [health officers] working in those PVTG villages beforehand so that we would be going there to give COVID vaccines to people”.

[FGD-06, CBOs, Chhattisgarh].

#### 3.4.3. Adaptations to Project Interventions

While project interventions were largely implemented as planned in both states, adaptations were made to improve effectiveness. Initially, the CBOs supported the governments by holding vaccination sessions at fixed times during the day, but these had low levels of turnout. After seeking community feedback and learning about how seasonal work affects people’s availability, CBOs and district health officials changed the times and locations of the sessions. They continued to ask for feedback on when to schedule vaccination sessions, which led to flexible timings and higher turnout. As a district officer who collaborated with the project said:

“Initially our team used to go for a session as per the official timings from 10 am to 4 pm. However, Tribal populations used to leave for their work early in the morning to the forest and come back after 5 pm, which usually meant that we would only be able to meet 1–2 people. Then it was discussed with the *patel* [village head] and *gayta pujari* [village priest] that the vaccination has to be arranged either early in the morning or after people come from work. Because the Tribal population would leave at 5 am, our teams used to be ready at 4:30 am to meet them and then again after 5 pm”.

[IDI-01, District health official, Chhattisgarh].

The CBOs established a call center to follow up with individuals who were due for their second dose, including those who had not received a vaccine certificate, and in Chhattisgarh, conducted door-to-door awareness activities on blocks with the lowest vaccination rates.

To manage staff attrition and accelerate vaccine uptake in low-coverage blocks, one district-level CBO team changed how it conducted community awareness activities. Rather than having teams conduct their own area-specific activities, the CBO implemented an “all hands-on deck approach.” The entire district-wide project workforce, including village ambassadors and block and district coordinators, conducted vaccine mobilization activities and sessions in every village across all blocks. To implement this adapted strategy, the CBO also provided government community health workers with transportation to and from vaccination sites and supported the delivery of vaccine boxes to the sessions in the villages.

As the focus shifted to increasing the uptake of the third or booster dose among Tribal populations, CBOs adapted their messages. Community health workers and CBOs organizing the *ratri chaupals* emphasized the urgency and advantage of receiving the third dose before it became a paid service. Many Tribal community members in both states acknowledged receiving this message. One stated:

“Regarding the third dose, health workers told us, ‘It’s government-provided and free now [like the first and second doses]. Later, you’ll have to go to Block Charma, and it will cost money”.

[FGD-03, Tribal population, Chhattisgarh].

In some districts, routine immunization days in the villages were an additional platform for promoting the third dose of the COVID-19 vaccine to caregivers. When there was high demand, an additional vaccination camp was organized the day after the *ratri chaupal*.

### 3.5. Maintenance

Based on interviews with project staff and district government officials, there were indications that the project’s interventions, especially its partnerships with community-based CBOs and leaders, had broad applicability in strengthening routine immunization and maternal health programs for Tribal populations. Funding was identified as a factor that sustained CBO support for COVID-19-related and other health-related programs at the district level, but there was broader acknowledgment that the involvement of communities and CBOs that worked with them was critical. Most immediately, continuous engagement with communities is crucial for identifying and adapting the most valuable and feasible project strategies.

Some government staff, particularly during the learning workshop, suggested focusing on adapting the project’s endeavors for other health programs, such as scheduling *ratri chaupal*, implementing vaccination sessions with flexible timings, and seeking collaboration across departments to develop line-lists of Tribal population members who needed services. They also recommended creating and sharing a directory of CBOs and emphasized the importance of engagement between CBOs and the government in district-level meetings to continue sharing feedback and information about Tribal populations and other high-priority groups.

## 4. Discussion

Our assessment suggests the project strategies were successful in reaching Tribal populations, creating vaccine awareness, and generating demand that led to uptake. While attributing all vaccination uptake solely to these strategies is challenging due to broader COVID-19 initiatives being implemented at the same time, the project had a crucial role in catalyzing COVID-19 vaccination efforts in the two states.

It is evident that collaboration with trusted community stakeholders increased vaccine awareness and demand. Organizations like SHGs that are deeply embedded within the Tribal community appeared to reinforce the benefits of vaccination and increase uptake. Furthermore, the integration of community members into project activities, particularly as village ambassadors, expanded the project’s reach in Tribal communities and conveyed information in culturally and linguistically familiar ways. These experiences underscore the significance of mitigating inequities by developing culturally responsive strategies and strengthening health systems so that they can be culturally responsive.

Recent studies conducted in India have similarly reflected on the importance of involving village leaders and partnering with local organizations to increase community engagement [24]. In particular, formal partnerships have been seen as facilitating community engagement [24] and shown to improve health practices and behaviors within the community [25]. For instance, partnerships with SHGs have been recognized as a promising strategy for reaching large numbers of women, including Tribal women, in India [26]. Research indicates that women residing in villages with active SHGs are more likely to access family planning methods, compared to those that lack such groups, highlighting the benefits of these partnerships [27]. Additionally, a recent social network analysis in rural India found that coordination between women’s self-help groups and local health systems improved when the village leader and the community health worker played central roles in the network [10]. This finding suggests that their involvement can be critical in facilitating information exchange between local health systems and community groups [10].

Our findings reinforced this, as collaboration with SHGs through village leaders and community health workers contributed to increasing vaccine awareness, access, and uptake.

Our study also highlights the importance of recognizing that community engagement efforts should not solely focus on conveying information, but also on soliciting input from communities about their needs and incorporating their feedback into the project. For example, platforms like *ratri chaupals*, where community members can provide feedback, express concerns, and actively participate, helped ensure that the project’s activities were culturally appropriate, community-driven, and responsive to specific needs and preferences. Integrating such community feedback mechanisms into programs can increase the reach of and demand for health services, specifically when aiming to improve routine immunization coverage among Tribal populations.

More broadly, there is a growing emphasis on prioritizing the involvement and collaboration of indigenous and tribal communities in designing solutions that can transform healthcare systems to become more responsive to their needs [28]. For example, in Canada, indigenous communities are beginning to actively participating in health policy-making processes [29]. Similarly, in the United States, initiatives have been launched to recruit health providers and community workers from Alaska Native and American Indian communities to better address their specific needs, and doing so has led to promising outcomes in increasing access to culturally responsive care and reducing the potential stigma associated with seeking care [30].

Equally important as involving and empowering Tribal community leaders in actively participating in health programs is creating a project environment that supports adaptability and flexibility. Key learnings from the project included collaborating with partners within Tribal communities and government, and using the information gathered through these collaborations to make better adaptations and decisions during program implementation, such as providing transportation for community health workers; improving data quality processes to ensure better reach; managing vaccine shortages by hiring additional vehicles; and supporting vaccine reallocation efforts.

While leveraging government program events such as *Aaapki Sarkar Aapki Dwar* (Your Scheme, Your Government at Your Doorstep) integrated vaccination with broader efforts, which reinforced the importance of vaccination to all populations, it was clear that vaccine ambassadors and village volunteers, along with community health workers, played a key role in rebuilding trust and communicating in a culturally appropriate manner among Tribal populations. Creating culturally appropriate and effective materials requires a deep understanding of cultural traditions and practices relating to health, wellness, and healing, which may differ from tribe to tribe with respect to such elements as using the herbs and remedies Tribal populations receive from their traditional healers in India [6] or connecting with one’s land to support healing processes as in American Indian/Alaskan Native communities [31], among others.

Several Tribal health programs and policies in India could be entry points for integrating the learnings from this case study. For instance, the National Health Policy [32], which recognizes the need to address inequities experienced by vulnerable populations such as Tribal groups, could incorporate interventions to promote Tribal community engagement in the design and implementation of healthcare programs and policies. Similarly, the hiring of community health workers and medical providers from within those communities can help to overcome language barriers and address the potential bias and discrimination experienced when seeking healthcare. The National Health Mission could also adopt such approaches to tailor health services and communications to promote cultural sensitivity and empathy when interacting with Tribal communities [24].

Community feedback mechanisms could be integrated into the Integrated Child Development Services, which works to improve the health and nutrition of women and children, and other health programs. As programs integrate these mechanisms, documenting the lessons will be critical for strengthening the evidence-base for what works and why, so others can learn and adapt and sustain such initiatives. Most of all, sustaining processes that involve communities in decision-making, respecting their needs, and collaborating with these communities will be imperative for ultimately increasing uptake and use of vaccination and health services among Tribal populations.

### Limitations

Key limitations to our study should be acknowledged. First, when we were unable to directly communicate with Tribal populations in their local languages, we relied on translators in a few instances, which may have hindered rapport building and caused us to miss nuances in their responses. Relatedly, we also recognize the role of interpreter bias in these situations, which may have influenced how certain comments were translated. Second, during our interviews, a few CBO staff members were new to their positions and may have lacked a comprehensive understanding of the project; we compensated for these gaps by speaking to other CBO supervisors based at the state level to gain a fuller understanding. Third, we had difficulty securing appointments with Tribal agency counterparts at the district level, so we sought insight through conversations with community leaders who interact with them. We attempted to increase the credibility of our findings by employing a triangulation approach, using multiple data sources to corroborate information (for example, speaking with multiple types of respondents), and engaging in respondent validation (member checking) by sharing our findings with individuals who were knowledgeable about the project and its context. A future quantitative assessment could provide additional data to complement this qualitative evaluation.

## 5. Conclusions

In conclusion, the project strategies appeared to be successful in effectively increasing vaccine awareness and demand among Tribal populations, and contributed to higher vaccine uptake. Although it is difficult to attribute all vaccination uptake solely to the project, due to concurrent COVID-19 vaccine initiatives, the project’s collaboration with trusted community stakeholders, particularly through village leaders, community health workers, and vaccine ambassadors, facilitated culturally appropriate communication and reinforced the benefits of vaccination within the community. The study also highlights the importance of community engagement and feedback mechanisms in designing and implementing healthcare programs that cater to the specific needs of Tribal communities. These mechanisms led to program adaptations that enabled the project to work towards its goals despite implementation challenges. These findings underscore the value of flexible, adaptable, and culturally sensitive approaches in healthcare programs. Partnerships with CBOs and community engagement strategies developed through this project can serve as a model for future health initiatives among Tribal populations. Sustaining such community-centered processes is key to enhancing the uptake of health services.

## Figures and Tables

**Table 1:** Key interventions to increase vaccine uptake implemented during the project period in Chhattisgarh and Jharkhand.

Strategy	Intervention	Frequency
Partner with district government and frontline community health workers to identify Tribal populations that need vaccination	District- and block-level CBO staff collaborated with district immunization officers and other government officials to identify individuals, including those from Tribal populations, who were due for COVID-19 vaccines. This initial step informed the development of microplans, which were essential for conducting door-to-door vaccination.	Initially, interactions were frequent (weekly/twice a week) to establish relationships, but later occurred fortnightly.
Collaborate with and integrate trusted community members into project activities	CBOs partnered with village heads to serve as community coordinators, who, in turn, made announcements about government- and project-sponsored vaccine awareness activities and vaccination sessions.	Initially, interactions were frequent (weekly/twice a week) to establish relationships, but later occurred fortnightly.
	CBOs trained community volunteers, sometimes referred to as vaccine ambassadors, to provide accurate information about the COVID-19 vaccine. These individuals were selected for their leadership qualities and ability to engage the community. Some volunteers were paid by the CBOs.	Training sessions were conducted when beginning the position, and refresher training sessions were held monthly.
	CBOs collaborated with local groups like *yuva mitan* clubs (youth groups) and women’s self-help groups (SHGs) to expand their outreach within the Tribal community.	Weekly
Implement interpersonal communication activities to allay vaccination concerns	Vaccine ambassadors conducted door-to-door visits, often in collaboration with community health workers, to create general awareness about the vaccine and mobilize community members to attend vaccination camps. Often, they also accompanied community members to the vaccination site.	Biweekly
	*Ratri chaupals* (evening community gatherings) were held to actively listen to Tribal populations’ concerns about vaccination and answer their questions. Implemented exclusively in the state of Chhattisgarh.	Weekly
Use government health- and non-health-sector platforms to increase awareness about the COVID-19 vaccines	At government events such as *Apki Yojana Apki Sarkar Apke Dwar* (“The Government is in Service to You”), the project set up vaccination stalls alongside government program booths and instructed staff at those booths to check vaccination status and refer people who were un- or under-vaccinated to the vaccination stall.	Project teams participated twice during these government events.
	Additionally, the project set up vaccination stalls at cultural and religious events like Durga Puja, Chhath Puja, local community gatherings, sports matches, and weekly markets to promote vaccination and vaccinate a broader audience, including Tribal populations.	Participation at cultural and religious events happened annually. Participation in community gatherings occurred on an ad hoc basis as these events arose.

Note: The interventions were implemented in both states unless otherwise stated.

**Table 2:** Description of the RE-AIM Framework.

Dimension	Definition
Reach	Whether, how, and why the interventions reached the Tribal populations
Effectiveness	Whether the interventions improved access to, demand for, and uptake of COVID-19 vaccines
Adoption	Whether, why, and how project staff and other stakeholders, such as the community-based CBOs and government, agreed to initiate/support the implementation of the interventions
Implementation	Whether, why, and how the interventions were delivered as intended, including adaptations made based on need and the evolving context
Maintenance	The sustainability of the interventions in the setting

Source: [20].

**Table 3:** FGD and IDIs, by respondent and state.

Respondent	Jharkhand	Chhattisgarh	Total
Tribal population			
FGD	10	7	17
IDI with particularly vulnerable Tribal group	10	11	21
CBO			
IDI with community-based CBO staff at the district, block, and community levels	3	3	6
FGD with district-, block-, and community-level CBO staff	2	3	5
Key stakeholder			
IDI with community-level stakeholder	5	7	12
IDI with district-level government stakeholder	9	11	20
IDI with state-level stakeholder	3	6	9
Total	42	48	90

**Table 4:** Summary of internal and external barriers to and facilitators of project implementation.

Barrier and Facilitators	Description of Actions Taken
Internal barriers	
Staff attrition	Quick hiring processes, when feasible; Learning opportunities from colleagues at various levels of experience facilitated knowledge sharing; Support and guidance from state-level project leadership.
Internal facilitators	
Regular meetings with state project leadership	Provided support to teams facing challenges through frequent meetings; Learning-by-doing approach guided program implementation and on-the-spot problem solving.
Hiring local staff	Helped to increase trust and accelerated implementation.
External barriers	
Government’s vaccine wastage policy	Sensitizing community members in advance of the vaccination session; Coordinating with other blocks or districts to identify additional eligible candidates helped ensure at least 10 individuals were present for vaccination.
Vaccine shortages	Transfer if excess supply to blocks or districts with low or no inventory.
Backlog of COVID-19 vaccine data entry into Co-WIN	CBOs supported data entry; CBOs hired temporary data entry staff at the block and district levels.
Inadequate transportation funds available to community health workers	CBOs provided transportation funds to frontline community health workers.
External facilitators	
“Whole of government” approach	Strong political will led to increased collaboration with all of the departments, which helped with the project’s implementation.
Strong partnerships with government and community and block health workers	The project’s relationships with Tribal populations, key leaders, and the health system offered valuable insights into program development and implementation; These relationships also facilitated ongoing feedback about the effectiveness of program strategies and highlighted areas where adaptations were needed.

## Data Availability

The data presented in this study are available on request from the corresponding author. The data are not publicly available due to the privacy concerns of the respondents.
